# Case report: Prolonged benefit of ESG401, a Trop2 antibody-drug conjugate, in endocrine-refractory hormone receptor-positive, HER-2 negative metastatic breast cancer

**DOI:** 10.3389/fonc.2024.1444431

**Published:** 2024-11-27

**Authors:** Jing Zhao, Fengbo Huang, Xia Xu, Yan Zhang, Xiaoyan Xing, Jian Huang, Fuming Qiu

**Affiliations:** ^1^ Department of Medical Oncology, Second Affiliated Hospital, Zhejiang University School of Medicine, Zhejiang University, Hangzhou, Zhejiang, China; ^2^ Key Laboratory of Tumor Microenvironment and Immune Therapy of Zhejiang Province, The Second Affiliated Hospital, Zhejiang University School of Medicine, Hangzhou, Zhejiang, China; ^3^ Cancer Institute, Key Laboratory of Cancer Prevention and Intervention, Ministry of Education, The Second Affiliated Hospital, Zhejiang University School of Medicine, Hangzhou, Zhejiang, China; ^4^ Department of Pathology, Second Affiliated Hospital, Zhejiang University School of Medicine, Zhejiang University, Hangzhou, Zhejiang, China; ^5^ Department of Pathology, Affiliated Jinhua Hospital, Zhejiang University School of Medicine, Jinhua, Zhejiang, China; ^6^ Shanghai Escugen Biotechnology Co., Ltd, Shanghai, China; ^7^ Cancer Center, Zhejiang University, Hangzhou, Zhejiang, China; ^8^ Department of Breast Surgery, Second Affiliated Hospital, Zhejiang University School of Medicine, Zhejiang University, Hangzhou, Zhejiang, China

**Keywords:** hormone receptor-positive, metastatic breast cancer, endocrine resistance, Trop2 antibody-drug conjugate, case report

## Abstract

Breast cancer (BC) remains a leading cause of cancer-related mortality in women, with hormone receptor-positive (HR+) tumors accounting for a significant proportion of cases. Despite advancements in endocrine therapy (ET), resistance remains a challenge in metastatic settings. The use of cyclin-dependent kinases 4/6 (CDK4/6) inhibitors in combination with endocrine therapy has notably improved survival. In China, when patients develop resistance to CDK4/6 inhibitors (CDK4/6i) or face financial constraints that prevent their use, chemotherapy becomes the standard treatment approach. This highlights an urgent need for effective treatments following CDK4/6i therapy. ESG401 is a novel trophoblast cell-surface antigen 2 (Trop2) directed antibody-drug conjugate (ADC) with promising preclinical and early clinical efficacy and safety data. We report a case of a 61-year-old female with HR+HER2- metastatic breast cancer (MBC) who developed resistance to fulvestrant and subsequent chemotherapy but achieved a durable partial response (PR) lasting more than 22.5 months following ESG401 treatment. This case underscores the potential role of Trop2-directed ADCs, such as ESG401, in overcoming endocrine resistance and providing meaningful clinical benefit in heavily pretreated patients with HR+/HER2- MBC. Furthermore, the patient’s exceptionally long clinical benefit distinguishes her from other patients receiving ESG401 treatment. Further exploration of the use of ESG401 in HR+HER2- MBC patients, as well as a deeper understanding of the characteristics of patients that may impact sustained efficacy, in expanded clinical trials is warranted.

## 1 Introduction

Breast cancer (BC) remains the most frequently diagnosed cancer, accounting for approximately 24.5% of all new cancer cases in 2020, and is the primary cause of cancer-related mortality in women (1). Hormone receptor-positive (HR+) tumors constitute 70-80% of all breast cancers (2). Due to the strong dependency of breast tumor development on the estrogen-estrogen receptor (ER) axis, estrogen suppression therapy and ER antagonists are the main treatments for hormone receptor-positive HER2-negative (HR+HER2-) metastatic breast cancer (MBC) (3). However, not all patients exhibit a favorable response to endocrine therapy (ET). Primary and acquired resistance to ET remains a challenge.

Several studies have revealed that endocrine therapy combined with cyclin-dependent kinase 4/6 inhibitors (CDK4/6i) has the potential to overcome resistance to ET and significantly improve survival rates (4–6). Additionally, alternative options such as PIK3CA inhibitors (7), mTOR inhibitors (8), AKT inhibitors (9), and emerging oral selective estrogen receptor degraders (SERDs) are gaining attention as later-line treatments (10). However, the accessibility of some of these treatments is limited in China. For patients who have exhausted endocrine therapy-based options, single-agent chemotherapy remains the standard of care. However, later-line chemotherapy is associated with limited effectiveness, reduced quality of life, and significant toxicity, highlighting the pressing unmet medical needs of these individuals.

ESG401 is a novel antibody-drug conjugate (ADC) comprising a humanized IgG1 monoclonal antibody targeting trophoblast cell-surface antigen 2 (Trop-2) linked to the topoisomerase I inhibitor SN-38 via a proprietary stable-cleavable linker with a drug-to-antibody ratio (DAR) of 8. The preliminary results of a phase I/II study in locally advanced/metastatic solid tumors revealed that ESG401 is safe and well tolerated with promising efficacy in heavily pretreated patients (11). Here, we report a patient with HR+/HER2- MBC who exhibited primary resistance to fulvestrant and had received multiple prior chemotherapy treatments but demonstrated a durable response lasting more than 22 months after treatment with ESG401. At the time of writing, the patient has maintained this response and is still receiving treatment, demonstrating excellent effectiveness and tolerability to ESG401. This case highlights the prolonged clinical benefit achieved by this patient following ESG401 treatment.

## 2 Case description

**Figure 1:**
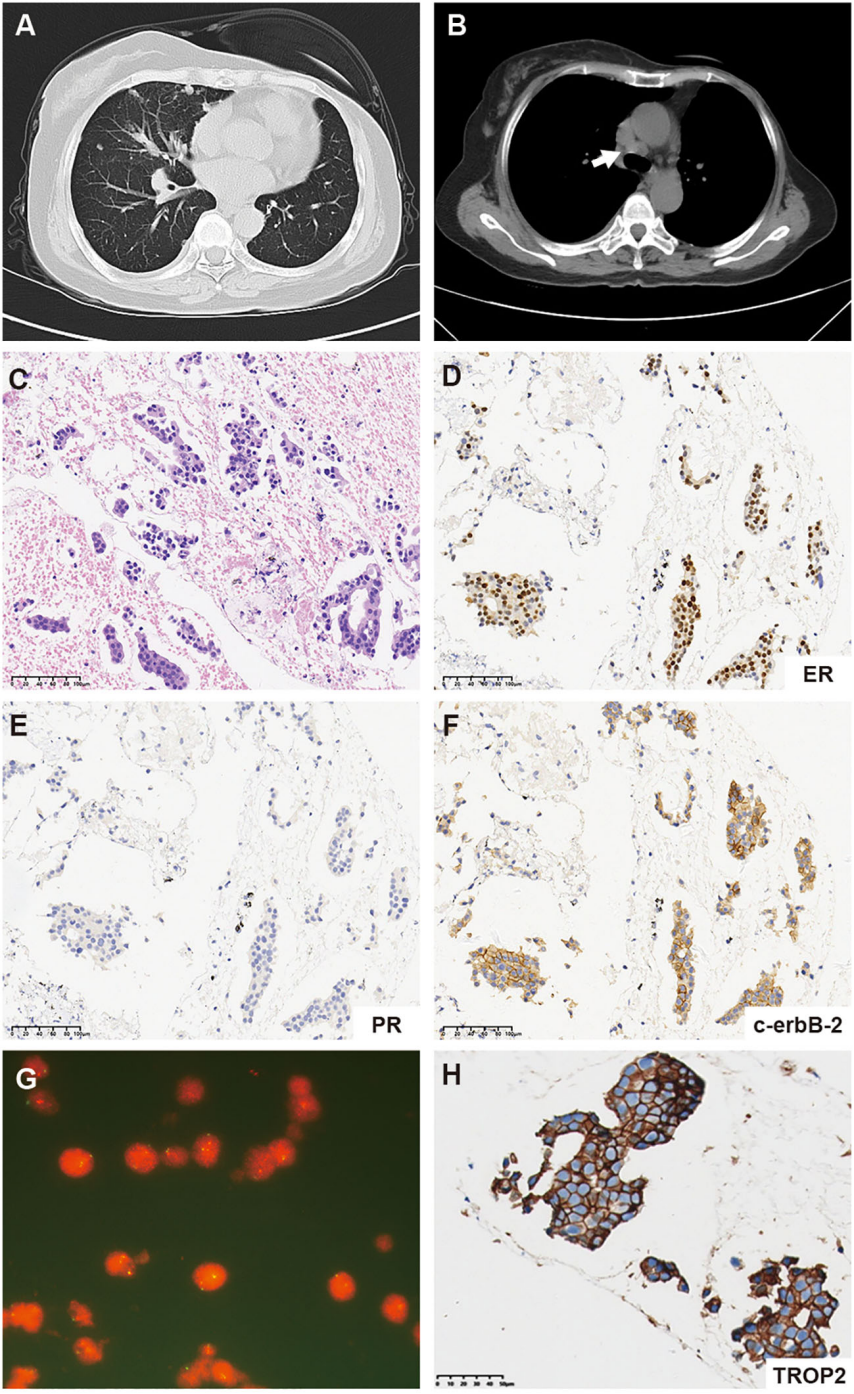
**(A)** Representative computed tomography (CT) scan images of the lung. **(B)** CT scan images of mediastinum lymph node metastases (white arrow). **(C)** Hematoxylin and eosin-stained (HE) section of a metastatic mediastinal lymph node (20×). **(D–F)** Immunohistochemistry (IHC) staining for ER, PR, and c-erbB-2 (HER2) expression in the metastatic mediastinal lymph node. **(G)** Fluorescence *in situ* hybridization (FISH) of the metastatic mediastinum lymph node revealed negative HER2 amplification. **(H)** Immunohistochemistry (IHC) staining for TROP2 expression in the metastatic mediastinal lymph node.

**Figure 2:**
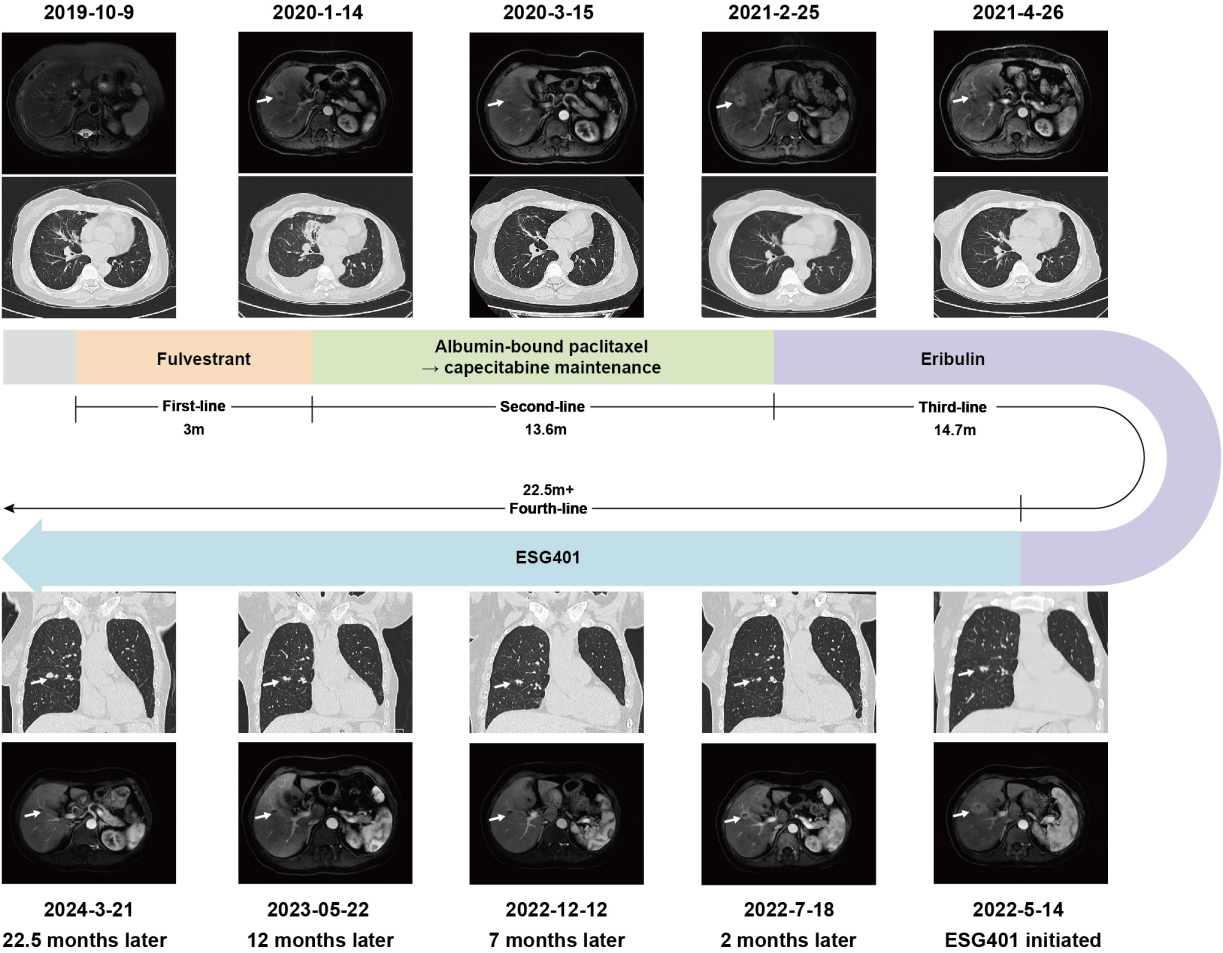
Imaging evolution of the patient treatment timeline and image efficacy evaluation.

In October 2019, a 61-year-old female patient presented to our hospital for the detection of multiple lung nodules by computed tomography (CT). She was diagnosed with lobular breast cancer (luminal B subtype) and underwent a modified radical mastectomy 5 years prior. She underwent adjuvant chemotherapy (epirubicin and cyclophosphamide followed by docetaxel) and received letrozole as adjuvant endocrine therapy until admission. She had a history of asthma, which was well controlled. There were no clinical symptoms or positive signs on physical examination. The chest CT revealed numerous pulmonary nodules, thickening of the bilateral pleural nodules, and enlarged lymph nodes in the mediastinum (**Figures 1A, B**). Endobronchial ultrasound-guided transbronchial needle aspiration of the mediastinum lymph node confirmed the recurrence of ER-positive, PR-negative, and HER2 2+/fluorescence *in situ* hybridization-negative breast cancer (**Figures 1C–G**). Metastases were not found in the brain, bones, or abdomen. She was diagnosed with metastatic lobular BC (lung, lymph node and pleural). The patient was advised to undergo CDK4/6 inhibitor-based endocrine therapy but was unable to afford it and declined the treatment at that time. Fulvestrant, a selective estrogen receptor down regulator, was administered. However, a CT scan performed 3 months later revealed significant growth in the lung nodules and a new mass in the liver (**Figure 2**), indicating primary resistance to endocrine therapy. Then, the patient underwent nine cycles of albumin-bound paclitaxel (200 mg, administered intravenously on Days 1 and 8 every 21 days) as second-line therapy, achieving a partial response (PR), followed by maintenance therapy with capecitabine. The progression-free survival (PFS) time was 13.6 months. Eribulin (2 mg, administered intravenously on Day 1 and Day 8, every 21 days) was then initiated as third-line therapy, resulting in another PR. Maintenance treatment with eribulin was continued for 14.7 months.

**Figure 3:**
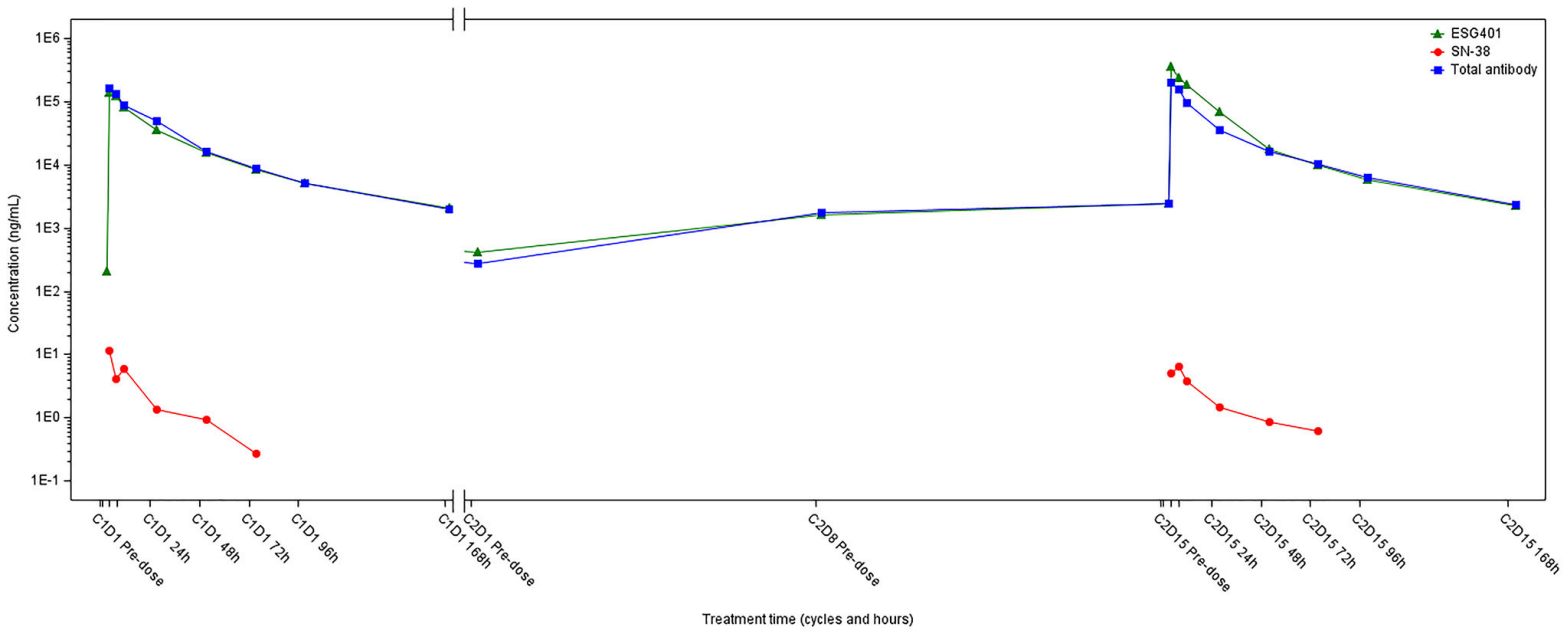
The plasma drug concentration-time curve of ESG-401, total antibody and SN-38 of the patient.

In May 2022, progression of the liver metastatic lesion was observed (**Figure 2**). The patient had an Eastern Cooperative Oncology Group (ECOG) performance status of 1 and was subsequently recommended to participate in a phase I/II clinical trial designed to evaluate the safety and antitumor effects of ESG401 (Shanghai Escugen Biotechnology Co., Ltd. Shanghai, China) in solid tumors (NCT04892342). ESG401 is a novel ADC with a humanized IgG1 antibody against the Trop2 antigen and the small molecule SN-38, which is a topoisomerase I inhibitor. An innovative stable and cleavable linker was used to combine the antibody to the payload with a DAR of 8. After providing informed consent, the patient received ESG401 treatment (12 mg/kg intravenously on Day 1, Day 8, and Day 15 every 28 days) on May 25, 2022. Trop2 status was assessed via IHC, revealing an H-score of 250, which was considered to be a high expression of Trop2(**Figure 1H**). After two treatment cycles, the CT scan showed a significant reduction in the size of the liver metastases, from 33 mm to 17 mm (a reduction of 48.5%), the overall efficacy of the patient was determined as PR according to RECIST 1.1. Four weeks later, the PR was confirmed. Subsequently, the patient’s lesions further decreased, with a total reduction in the sum of the longest diameters of the target lesions of 52.6%. At the time of writing (May 2024), the patient had sustained a beneficial response for 23.6 months. The latest CT scan from March 21, 2024, revealed a sustained PR for the liver mass. Although a lung nodule has gradually gotten larger, the overall assessment of efficacy is still a PR. The pharmacokinetic profile of this patient indicates that, irrespective of whether they received a single or multiple dose of ESG401, the concentrations of both the total antibody and the intact ADC remained similar. However, the concentration of SN-38 was significantly lower compared to the total antibody and ESG401, with a maximum concentration (Cmax) of 6.5 ng/ml and an area under the curve (AUC) of 136 ng·h/ml. The pharmacokinetics (PK) profiles of total antibody and ADC drug are very similar, indicating that ESG401 is very stable in circulation (**Figure 3**). The patient has experienced very mild and easily manageable adverse reactions, including grade 2 neutropenia, grade 1 diarrhea, and anemia, during ESG401 treatment. The patient maintains an excellent performance status and has a high quality of life.

## 3 Discussion

Endocrine resistance, which refers to resistance to estrogen or ER suppression, remains a significant hurdle for patients with HR+/HER2- BC. For MBC, primary endocrine resistance is defined as disease progression during the first 6 months of first-line ET (12). The mechanism is intricate and potentially linked to modifications in the estrogen receptor pathway (e.g., ESR1 mutations) or upstream signaling pathways of growth factors (e.g., the PI3K/Akt/mTOR pathway) (13). In the last two decades, there have been notable advancements in the discovery and approval of novel medications targeting the ER and upstream signaling pathways. Among the drugs available in China, CDK4/6i combined with fulvestrant is the standard treatment for patients who have progressed on aromatase inhibitors (AIs) (14). In our specific case, the patient opted against receiving CDK4/6i treatment due to financial limitations and subsequently experienced rapid progression while on fulvestrant monotherapy. In addition to chemotherapy, the mTOR inhibitor everolimus in combination with exemestane is a favorable option, potentially prolonging PFS in patients who are refractory to nonsteroidal AIs (8). Nevertheless, the relatively high toxicity profile of everolimus limits its utilization. Although currently unavailable in China, PI3K and AKT inhibitors have shown potential to improve prognosis in HR+/HER2- BC patients with activation of the PI3K/AKT/mTOR pathway, providing an alternative strategy to overcome endocrine resistance (7, 9, 15).

ADCs are a class of targeted therapies that consist of monoclonal antibodies conjugated with cytotoxic drugs. The monoclonal antibody component enables specific recognition and binding to cancer cell surface antigens, while the cytotoxic drug payload delivers a potent anticancer effect directly to tumor cells. Trastuzumab emtansine (T-DM1) is the first approved ADC that targets HER2 (16). Compared with T-DM1, trastuzumab deruxtecan (T-DXd), another ADC that targets HER2, has been shown to improve survival outcomes in patients with HER2-positive MBC (17); it also improves survival outcomes in patients with MBC with low HER2 expression compared to those receiving standard chemotherapy (18). Trop-2 is another target of interest in breast cancer. It was initially identified in 1981 as a protein prominently present on the surface of trophoblast cells (19). However, subsequent studies revealed its intricate involvement in cancer cell processes, including growth, proliferation, migration, invasion, and survival (20). In breast cancer, expression of the TROP-2 gene has been identified across all subtypes, with particularly elevated levels observed in HR+ HER2-negative and triple-negative breast cancer (TNBC) compared to HER2-positive disease (21). Elevated TROP-2 expression was correlated with poorer survival outcomes (22).

Sacituzumab govitecan (SG) is the first ADC directed against Trop-2 and was recently approved in China for patients with metastatic triple-negative breast cancer (mTNBC) who have undergone two or more lines of chemotherapy. SG consists of a humanized anti-Trop-2 monoclonal antibody linked to SN-38 via a hydrolysable CL2A linker (23). Datopotamab deruxtecan (Dato-DXd) is another Trop2-targeted ADC that combines a humanized anti-TROP-2 IgG1 monoclonal antibody with a topoisomerase I inhibitor through a cleavable tetrapeptide linker, achieving a DAR of 4. Although these three drugs are all Trop2-targeting ADCs, there are certain differences in their molecular composition. A comparison of these three drugs can be found in **Table 1**.

**Table 1:** Comparison of molecular composition of Three Trop2 Directed Antibody-Drug Conjugates (ADCs).

Product	ESG401	Trodelvy	DS-1062
Antibody	Sacituzumab	Sacituzumab	Datopotamab
Payload	SN-38	SN-38	DXd
Linker	MC-VC-PAB	CL2A	GGFG
Type of Linker	Enzyme dependent	pH-dependent	Enzyme dependent
DAR	8	7.6	4

In HR+HER2- MBC patients resistant to endocrine therapy, sacituzumab govitecan significantly increased the median progression-free survival (mPFS) (5.5 months vs. 4.0 months) and median overall survival (mOS) (14.4 months vs. 11.2 months) compared to that with conventional chemotherapy (24). However, safety analysis revealed a greater incidence of adverse events (AEs) in the SG group, with notable neutropenia, diarrhea, nausea, vomiting, alopecia, and fatigue. The hydrolysable CL2A linker, which is designed for extracellular hydrolysis, leads to an earlier release of free SN-38 into the circulation, leading to a higher peak cytotoxin concentration, a higher exposure and potentially contributing to an increase in AEs (25).Compared to investigator’s choice of chemotherapy, datopotamab deruxtecan (Dato-DXd) led to significant improvement in PFS (6.9 months vs. 4.9 months) and presented a favorable safety profile in a phase III trial for HR+/HER2- MBC (26). These studies demonstrate that Trop2-directed ADCs offer substantial promise as a novel therapeutic approach for HR+/HER2- MBC.

An optimal linker for an ADC should possess adequate stability to transport the payload to the desired site while also exhibiting sufficient lability to release an effective quantity of payload either within the tumor or in the tumor microenvironment (TME) (27). ESG401 is a novel ADC directed toward Trop2 that utilizes an innovative stable cleavable linker designed to conjugate SN38 to a humanized monoclonal antibody targeting Trop2 with a DAR of 8. In the design of ESG401, it is expected that, due to the increased stability of the linker, the intact ADC molecule will be less likely to dissociate in systemic circulation, resulting in lower systemic exposure of the free payload. It showed that the longer half-life of intact ADC in the patient than SG (49.0 vs.23.4 hours), with lower exposure of SN38, reflected in the ratio of SN38 to ADC (0.002% vs. 0.07%) (28). Furthermore, the pharmacokinetic evaluation of the patient, the same as other patients from the trial, exhibited notably reduced maximum concentration (Cmax) levels (6.5 vs. 98.0 ng/ml) and area under the curve (AUC) values (136 vs. 3696 ng·h/ml) for SN-38 when contrasted with the published pharmacokinetic profile of SG (28). These PK characteristics suggest that ESG401 is more stable in circulation. This may mechanistically explain the patient’s milder off-target toxicities, such as neutropenia, nausea, and diarrhea. The toxicity profile of ESG401 was moderate, as reported, the most frequent treatment-related adverse events (TRAEs) of Grade 3 or higher were leukopenia (29%) and neutropenia (31%) (11). There were no Grade 3 or higher events of thrombocytopenia, diarrhea, skin rash, or oral mucositis. The clinical trial protocol for participating patients also provides detailed guidelines on the management of potential adverse events (AEs), including intervention thresholds and any necessary dose modifications due to toxicity (**Supplementary Table 1**).

This profile is attributed to the stability of the ADC molecule conferred by the proprietary linker of ESG401 and the reduced release of free payload in the system, thereby minimizing off-target toxicity. The cleavable linker and membrane-permeable payload SN-38 enable it to exert a “bystander effect” (29). In tumor-bearing mouse models, compared with tumors treated with SG, tumors treated with ESG401 had higher levels of and longer exposure to both free SN38 and total SN38 within tumor tissues. The combination of decreased serum release and increased exposure of SN-38 within tumor tissues indicated the reliable efficacy and exceptional safety of ESG401 in an animal model. Initial findings from a phase I/II clinical trial assessing the safety, tolerability, pharmacokinetics, and antitumor effects of ESG401 in patients with locally advanced or metastatic solid tumors have been reported (11). Thirty-five heavily treated patients (with a median of 4 (2-10) prior lines of treatment) were enrolled. Among 33 patients assessed for efficacy, the ORR and DCR across various dosage regimens were 36.4% (12/33) and 63.6% (21/33), respectively. Specifically, in a subgroup of 13 patients receiving therapeutically relevant doses of HR+/HER2- MBC, the ORR and DCR were 62% (8/13) and 77% (10/13), respectively. These promising efficacy signals suggest a favorable treatment effect of ESG401 for HR+/HER2- MBC, which is consistent with the case of this patient with exceptionally long clinical benefit that we have reported. In addition, an open-label, randomized, active-controlled, multicenter, Phase III study (NCT06383767) of ESG401 versus Investigator’s Choice Chemotherapy (ICC) is currently underway. This trial involves patients with HR+/HER2- locally advanced or metastatic breast cancer who have progressed during endocrine therapy, are unsuitable for endocrine treatment, and have received at least one prior line of systemic chemotherapy. The study also aims to validate the findings from this case study in a larger patient cohort. In our case, the patient who rapidly progressed on fulvestrant and subsequently received two additional lines of chemotherapy achieved a significant PR following treatment with ESG401. This favorable response lasted for more than 22.5 months with excellent tolerance and good quality of life, exceeding the duration achieved with previous chemotherapy; this response period was also much longer than the published mPFS from studies using SG (5.5 months) and Dato-Dxd (6.8 months) in the same indication population. This prolonged response may be attributed to the unique mechanism of action of ADCs with cytotoxic payloads, which may unaffected by resistance mechanisms typically associated with endocrine therapy. Additional contributing factors include high Trop2 expression (H-score: 250). In the ASCENT trial, a more favorable overall survival (OS) trend was observed in patients with higher Trop2 expression (30). Furthermore, low tumor burden, good performance status, and the demonstrated efficacy of ESG401 also likely contributed to the therapeutic benefit. Some possible underlying unveiled reasons may have an impact as well. Further exploration of these factors that may contribute to the patient’s prolonged duration response will help to further elucidate the mechanism of drug action and provide meaningful guidance for the treatment of such patients. Moreover, diverse reactions to ESG401 were observed in hepatic and pulmonary lesions, emphasizing the heterogeneous nature of tumors. Variability in TROP2 expression across distinct metastatic loci in breast cancer has been documented (31). Re-evaluation through lung metastasis re-biopsy at the onset of progressive disease (PD) could offer valuable insights into the resistance mechanisms of ESG401, thus guiding subsequent therapeutic strategies. Furthermore, the patient’s HER2 status is 2+. Trastuzumab-deruxtecan (T-DXd), an ADC targeted to HER2, could be an alternative treatment strategy. In DESTINY-Breast04 trial, T-DXd demonstrated improved PFS and OS benefit in advanced HER2-low breast cancer compared to chemotherapy of physician’s choice (18).

The limitations of this case study include the absence of CDK4/6 inhibitor therapy, which has become a cornerstone in treating luminal-like metastatic breast cancer. This patient did not receive CDK4/6 inhibitors due to financial constraints and personal choice. In the clinical trial (NCT04892342) she participated in, 78% of the 65 HR+/HER2- patients had prior exposure to CDK4/6 inhibitors. Among the 58 efficacy evaluable patients, the ORR was 27.7% for those with prior CDK4/6 inhibitor treatment and 54.5% for those without (unpublished internal data). These results were derived from a retrospective analysis, which may be influenced by baseline imbalances and the small sample size, making it unclear whether prior CDK4/6 inhibitor use affects the efficacy of ESG401. Additionally, the patient did not undergo molecular testing, so her suitability for targeted therapies, such as PIK3CA/AKT/mTOR inhibitors, remains unknown. Further studies are needed to explore the potential interaction between gene-targeted therapies and TROP2 ADCs in a larger cohort. Another important point is that, although the patient experienced only mild and easily manageable adverse reactions, including grade 2 neutropenia, grade 1 diarrhea, and anemia, during over 22.5 months of ESG401 treatment, this Phase I study did not include standardized quality-of-life assessments or patient-reported outcome measures. Therefore, any improvement in the patient’s quality of life has not been objectively demonstrated. In the planned Phase III study, quality-of-life assessments will be included as one of the secondary endpoints.

In conclusion, for patients with endocrine-refractory HR+/HER2- MBC, Trop2-directed ADCs represent an optimal choice for later lines of therapy. Enhanced ADC engineering holds significant promise for maximizing both the efficacy and safety of these agents. ESG401 has demonstrated favorable tolerability with promising signs of efficacy in heavily pretreated patients. Further investigations through expanded clinical trials in HR+/HER2- MBC, as well as a deeper understanding of the characteristics of patients that may impact sustained efficacy, are warranted.

## Data Availability

The original contributions presented in the study are included in the article/**Supplementary Material**, further inquiries can be directed to the corresponding author/s.
