# Supplementary material for: Case report: Prolonged benefit of ESG401, a Trop2 antibody-drug conjugate, in endocrine-refractory hormone receptor-positive, HER-2 negative metastatic breast cancer
Source: Front Oncol. 2024 Nov 27;14:1444431. doi: 10.3389/fonc.2024.1444431 (PMC11631905; doi:10.3389/fonc.2024.1444431)
Supplement: Supplementary file 1 [file Table1.docx]

**Supplementary Table 1. Dose Modification Guidelines for ≥Grade 3 Events Considered Related to Study Treatment**

| **CTCAE Grade** | **Times** | **Dose Modification** |
| --- | --- | --- |
| **Hematologic toxicitites** | | |
| - Grade 4 neutropenia lasting ≥5 days - Febrile neutropenia (defined as absolute neutrophil count [ANC] <1000/mm3 with a single temperature of 38.3℃ or a sustained temperature of 38℃ for >1 hour) - Grade ≥3 hematologic toxicities with the duration of remission to grade 1 toxicity exceed 1 week but does not exceed 3 weeks | First | Restart study treatment with 25% reduction of dose |
|  | Second | Restart study treatment with 50% reduction of dose |
|  | Third | Permanently discontinue study treatment |
| - Grade ≥3 hematologic toxicities with the duration of remission to grade 1 toxicity exceed 3 weeks | First | Permanently discontinue study treatment |
| **Non-hematologic toxicitites** | | |
| - Grade 4 or higher toxicity - Grade 3 toxicities irrespective of duration, (except for laboratory abnormalities with no clinical significance, diarrhea, nausea, vomiting, or rash, that improve to Grade 3 or lesser severity within 2 days of the institution of supportive care) - Grade ≥3 non-hematologic toxicities with the duration of remission to grade 1 toxicity exceed 1 week but does not exceed 3 weeks | First | Restart study treatment with 25% reduction of dose |
|  | Second | Restart study treatment with 50% reduction of dose |
|  | Third | Permanently discontinue study treatment |
| - Grade ≥3 non-hematologic toxicities with the duration of remission to grade 1 toxicity exceed 3 weeks | First | Permanently discontinue study treatment |
